# Performance of Claims-based Dementia Algorithms in Medicare Fee-for-service and Medicare Advantage Populations

**DOI:** 10.1093/geroni/igaf122.4238

**Published:** 2025-12-31

**Authors:** You Wu, Chan Mi Park, Lan Luo, Xiecheng Chen, Eunji G Kim, Sandra Shi, Dae Hyun Kim, Ellen McCarthy

**Affiliations:** Harvard T.H. Chan School of Public Health, Boston, Massachusetts, United States; Hebrew SeniorLife, Harvard Medical school, Boston, Massachusetts, United States; Marcus Institute for Aging Research, Boston, Massachusetts, United States; Hebrew Rehabilitation Center, Roslindale, Massachusetts, United States; Marcus Institute for Aging Research, Boston, Massachusetts, United States; Hebrew SeniorLife, Boston, Massachusetts, United States; Hebrew SeniorLife, Boston, Massachusetts, United States; Hebrew SeniorLife, Boston, Massachusetts, United States

## Abstract

Claims-based dementia algorithms have not been evaluated in the Medicare Advantage (MA) population, a critical gap given the rapid growth of MA and concerns about data completeness. Using community-dwelling participants from the National Health and Aging Trends Study (NHATS) Round 7 (2017; n = 5,058), Round 9 (2019; n = 4,048), and Round 11 (2021-2022; n = 3,109), we validated the Bynum Standard and Chronic Condition Warehouse (CCW) dementia algorithms against NHATS classification for probable dementia. The prevalence of probable dementia was similar between MA (8.2%-9.1%) and FFS (8.1%-8.5%) across rounds. Overall, both algorithms had comparable performance in MA vs FFS populations, demonstrating high specificity (>97.9%) and negative predictive value [NPV] (>92.9%). The Bynum algorithm had similar sensitivity between MA and FFS in Round 7 (30.2% vs. 29.1%) and 11 (25.6% vs. 23.9%), but statistically significant and clinically meaningful lower sensitivity in MA for Round 9 (25.0% vs. 36.4%). However, positive predictive value [PPV] was similar between MA (62.7%-70.6%) and FFS (62.1%-70.4%) in all rounds. Comparatively, the CCW algorithm had statistically significant and clinically meaningful lower sensitivity in MA than FFS for Round 9 (30.8% vs. 46.9%) and 11 (35.0% vs. 49.3%), yet similar sensitivity for Round 7 (40.4% vs. 42.2%). The PPV in MA and FFS varied across rounds, from Round 7 (76.3% vs. 71.2%) to 11 (68.5% vs. 76.4%). Claims-based dementia algorithms have lower or comparable sensitivity in MA vs. FFS, with similarly moderate PPV and consistently high specificity and NPV. Researchers should consider these trade-offs when selecting algorithms for MA and FFS.

